# Biochemical characterization and evaluation of cytotoxicity of antistaphylococcal chimeric protein P128

**DOI:** 10.1186/1756-0500-5-280

**Published:** 2012-06-08

**Authors:** Shilpa E George, Ravisha Chikkamadaiah, Murali Durgaiah, Amruta A Joshi, Ullas P Thankappan, Shampur N Madhusudhana, Bharathi Sriram

**Affiliations:** 1 Gangagen Biotechnologies Pvt Ltd., No. 12, 5th Cross, Raghavendra Layout, Tumkur Road, Yeshwantpur, Bangalore, 560 022, India; 2 Current address: Department of Neurovirology, National Institute of Mental Health & Neurosciences, Bangalore, 560029, India

## Abstract

**Background:**

Antibiotic resistant *S. aureus* infection is a global threat. Newer approaches are required to control this organism in the current scenario. Cell wall degrading enzymes have been proposed as antibacterial agents for human therapy. P128 is a novel antistaphylococcal chimeric protein under development against *S. aureus* for human use which derives its bacterial cell wall degrading catalytic endopeptidase domain from ORF56, the Phage K tail-structure associated enzyme. Lead therapeutic entities have to be extensively characterized before they are assessed in animals for preclinical safety and toxicity. P128 is effective against antibiotic resistant strains as well as against a panel of isolates of global significance. Its efficacy against *S. aureus in vivo* has been established in our lab. Against this background, this study describes the characterization of this protein for its biochemical properties and other attributes.

**Results:**

We evaluated the requirement or effect of divalent cations and the metal ion chelator, EDTA upon biological activity of P128. As the protein is intended for therapeutic use, we tested its activity in presence of body fluids and antibodies specific to P128. For the same reason, we used standard human cell lines to evaluate cytotoxic effects, if any.

The divalent cations, calcium and magnesium at upto 25 mM and Zinc upto 2.5 mM neither inhibited nor enhanced P128 activity. Incubation of this protein with EDTA, human serum, plasma and blood also did not alter the antibacterial properties of the molecule. No inhibitory effect was observed in presence of hyper-immune sera raised against the protein. Finally, P128 did not show any cytotoxic effect on HEp2 and Vero cells at the highest concentration (5 mg/mL) tested.

**Conclusions:**

The results presented here throw light on several properties of protein P128. Taken together, these substantiate the potential of P128 for therapeutic use against *S. aureus*. Further development of the protein and conduct of preclinical safety studies in animals is warranted.

## Background

Current treatment for staphylococcal infections face multiple challenges including spread of highly virulent strains in the hospital and community environments. Novel therapeutic agents and modalities are becoming a necessity to combat the often invasive infections by these strains [1,2].

In *S. aureus* and other Gram-positive bacteria, where the outer membrane is absent, the multilayered peptidoglycan or murein, commonly referred to as the cell wall, represents the limit of the cell. Peptidoglycan is an important component in these bacteria, conferring strength and rigidity to the cell, allowing growth and division, maintaining cell shape, and protecting against osmotic lysis [3]. The peptidoglycan therefore presents an excellent target for antimicrobial action. Murein degrading enzymes that are capable of hydrolyzing the various carbohydrate or protein linkages of the peptidoglycan layer bring about cell death. Such enzymes are produced in eukaryotic and prokaryotic organisms [4-6]. The most appropriate sources of such enzymes would be bacteriophages, which are viruses that prey on bacteria and have co-evolved with their host. P128 is a novel chimeric bacteriophage derived protein that is under development in our laboratory for human therapy against antibiotic resistant staphylococci. P128 has shown potent *in vitro* bactericidal activity against many staphylococci, including methicillin-resistant *S. aureus* (MRSA) and has been efficacious as a topical gel in reducing MRSA nasal carriage in an animal model [7]. Efficacy of P128 when administered parenterally, is a key aspect that is currently under investigation.

Various systemic components and prevailing conditions or microenvironments could potentially impact the structure, stability, availability and thereby the biological activity of P128. In case of antibiotics, plasma protein binding is often associated with a loss in antibacterial activity. Loss due to plasma protein binding is shown to be upto 97% in case of Fusidic acid [8]. This loss is upto 90% for Daptomycin and the Minimum Inhibitory Concentration (MIC) of this antibiotic is adversely affected in the presence of human serum with associated clinical failures [9]. In case of antimicrobial peptides (AMPs), it is known that various defensins are partly or completely antagonized by high salt conditions or the presence of plasma proteins *in vitro*[10,11]. It is also known that the activity of cationic antimicrobial peptides, such as neutrophil defensins is influenced by various microenvironmental conditions [12].

Cell wall degrading enzymes are of significantly higher molecular size compared to AMPs. However, they have to face similar challenges in physiological environments that could potentially affect protein folding and stability, or cause inactivation or inhibition of enzymatic activity. Inorganic ions, especially metal ions such as Ca^2+^, Mg^2+^ and Zn^2+^, have been shown to modulate the activity of bacterial cell wall degrading enzymes [13,14]. The likelihood of degradation of such proteinaceous antibacterials by proteases and neutralization by the immune system [15] cannot be ignored.

Considering the heterogeneity of biological fluids, understanding the impact of the physiological and biological components in such complex matrices on the activity of P128 would be critical and forms the basis of this work. Bactericidal activity of P128 is tested under various conditions including presence of divalent cations (Ca^2+^, Mg^2+^ and Zn^2+^), EDTA, hyper-immune sera; human serum; plasma; and whole blood.

A key milestone that lead molecules have to reach, for further development as drug candidates, is that they have to be declared ‘safe’ for use [16]. The major challenge that overrides the potent and broad-spectrum activities of many antimicrobial peptides is in fact, the cytotoxicity they may pose [17,18]. The predictive value of *in vitro* cytotoxicity tests that reflect ‘basal’ cytotoxicity or adverse effects on basic functions common to all cells, is well-recognized [19]. Understandably, such assays are routinely applied to the evaluation of pharmaceutical molecules as part of the drug discovery process. P128 has been evaluated in the present work for cytotoxicity on two mammalian cell lines.

## Methods

### P128 expression and purification

P128 protein was expressed and purified as described previously [20].

### Bacterial strains

All strains were cultured in Luria Bertani (LB) broth at 37°C on a rotary shaker (200 rpm). MRSA clinical isolate COL and mupirocin-resistant community-associated MRSA USA300 were used for the bactericidal activity assays. Cell culture media were obtained from HiMedia labs (India).

### Bactericidal activity assay

The bactericidal activity of P128 was assessed by a CFU reduction assay. Briefly, cultures of *S. aureus* were grown in LB broth until absorbance at 600 nm (A_600_) reached 1.0, and then diluted in LB broth to obtain 1x10^8^ cells/ml. For all assays, 1x10^8^ cells/ml were used, unless otherwise stated. Cells (~1x10^7^ in 100 μl) were treated with purified protein at specified concentrations in 100 μl volume and incubated at 37°C, 200 rpm for 1 h. The residual viable bacterial cells (colony forming units; CFUs) were enumerated by serial dilution and plating on LB agar plates. Dose–response of P128 was evaluated using this assay with varied cell numbers ranging from ~10^8^ to ~10^1^ cells/ml of *S. aureus* USA300 treated with purified P128 in the concentration range of 0.25 – 10 μg/ml.

### Effect of divalent cations and EDTA as modulators of P128 activity

The effect of calcium, magnesium and zinc ions on P128 activity was determined at 37°C in saline, containing different concentrations of Calcium Chloride or Magnesium Chloride or Zinc Acetate.

2 μg of purified P128 was incubated in 10, 20, and 50 mM CaCl_2_ (Sigma, USA) or MgCl_2_ (HiMedia) at 37°C for 30 min. Similarly, 2 μg of P128 was incubated in 0.5, 1, 2 and 5 mM Zinc Acetate (Merck, India) at 37°C for 30 min. This was then tested for bactericidal activity on *S. aureus* USA300 as described. The final salt concentrations in the assay were 5, 10 and 25 mM of CaCl_2_ or MgCl_2_ and 0.25, 0.5, 1 and 2.5 mM Zinc Acetate.

The effect of EDTA on P128 activity was determined at 37°C in saline, containing different concentrations of K_2_ EDTA. 2 μg of purified P128 was incubated in 1.25, 2.5, 5, 10 and 20 mM K_2_ EDTA (Merck, India) at 37°C for 30 min. This was then tested for bactericidal activity on *S. aureus* USA300 as described. The final salt concentrations in the assay were 0.625, 1.25, 2.5, 5 and 10 mM K_2_ EDTA.

### P128 activity in human plasma, serum and whole blood

Bactericidal activity of P128 on *S. aureus* was tested in presence of human biological matrices using the CFU reduction assay format. To prepare serum, 6 ml of whole human blood was collected using a pyrogen free syringe and allowed to clot at ambient temperature. Tubes were centrifuged at 2300 x g for 5 min and serum collected. For whole blood, 6 ml of blood was collected and K_2_EDTA (Merck, India) was added at a concentration of 1.5 mg/ml. Human plasma was from a commercial source.

2 μg of purified P128 was diluted into 100 μl plasma, serum or whole blood from a 1 mg/ml stock (final assay concentration - 10 μg/ml). 1×10^7^ cells of *S. aureus* COL or USA300 were resuspended in 100 μl of plasma, serum or whole blood (final assay concentration - 5×10^7^ cells/ml) and treated with P128 that was pre-incubated at ambient temperature for 20 min in the corresponding matrix. Cells incubated in serum, plasma or whole blood without addition of P128 served as controls. Residual CFU were enumerated at the end of 1 h incubation at 37°C.

### Effect of hyper-immune sera on P128 activity

P128-specific antibodies were tested for the ability to neutralize the activity of P128. New Zealand White rabbits were immunized with purified P128 using a standard protocol comprising a primary immunization with complete Freund’s adjuvant and three boosts in incomplete Freund’s adjuvant. Enzyme-linked immunosorbent assay (ELISA) titers were>100,000 (reciprocal of the dilution of the inflection point in a typical best-fitted sigmoid curve).

The assay procedure was modified from that described previously [21]. P128 in 0.85% saline (100 μl) was diluted from 100 μg/well – 0.09 μg/well through 11 wells in a microtiter plate. Ten microliters of either hyper-immune rabbit serum, pre-immune serum, or saline was added to each well and incubated at 25°C for 15 min. An equal volume (100 μl) of *S. aureus* USA300 containing 1x10^8^ cells (final assay concentration - 5x10^8^ cells/ml) was then added to each well. After 30 min at 25°C, 50 μl from each assay well was plated out and residual CFU enumerated. Cells incubated in saline and treated with P128 prepared in saline served as the control for biological activity at each test concentration of the protein in the assay. Cells incubated in saline, pre-immune or hyper-immune sera without the addition of P128 served as cell controls.

### *In vitro* cytotoxicity of P128

The cytotoxicity of P128 was evaluated on HEp2 and Vero cell lines, using MTT (3-(4,5-dimethylthiazol-2-yl)-2,5-diphenyltetrazolium bromide) assay [22]. HEp2 (ATCC Cat. No.CCL-23, Human epithelial carcinoma) and Vero (ATCC Cat. No. CCL-81, African monkey kidney) were purchased from National Centre for Cell Sciences, Pune, India and were maintained in Eagle’s Minimum Essential Medium (EMEM) (Sigma, USA) supplemented with 10% Fetal Bovine Serum (European Grade FBS, Biological Industries, Israel) and 100 U/mL of ampicillin and 100 μg/mL of streptomycin (Sigma, USA), in a 37°C incubator (NuAire, USA) under an atmosphere of 5% CO_2_.

Briefly, 1×10^5^ cells were seeded per well in a 96-well flat-bottom microtitre plate (CellStar, Greiner), in 100 μL of complete growth medium and the plate incubated at 37°C for 24 h. After 24 h, fresh growth medium was replenished in the wells. P128 was diluted from an original stock to obtain concentrations ranging from 0.078 to 5 mg/mL, in serum-free EMEM, and 100 μL of each solution was added to respective wells. A 0.5 mg/mL solution of mitomycin C (Sigma, USA; prepared in EMEM) was employed as positive control, and cells treated with 100 μL of EMEM served as negative controls. Each group was evaluated in quadruplicates and the whole experiment repeated twice. The cells were incubated with the solutions for a period of 24 h, followed by removal of the solutions and replenishment of fresh growth medium. Twenty μL of a stock solution of MTT (Sigma, 5 mg/mL in double distilled water) was added per well and the plate incubated in the dark for 4 h at 37°C. This was followed by removal of the contents of the wells and dissolution of the formazan crystals by addition of 150 μL/well of dimethyl sulfoxide (Sigma, USA) solution. Absorbance values at 590 nm were obtained with the help of a UV–vis spectrophotometer (TECAN) within 15 min. The percentage cell viability in each group was calculated in relation to that of the untreated control cells.

### Data analysis

A minimum of two experiments were performed for each CFU reduction assay & cytotoxicity assay. Results were obtained as means and standard deviations. Statistical significance was evaluated by one-way analysis of variance (ANOVA) followed by Tukey-Kramer Multiple Comparisons Test wherever appropriate. A value of P<0.05 was considered to be significant. Statistical analysis was done using Microsoft Excel® and GraphPad InStat trial version from GraphPad Software.

## Results and discussion

It is well-established that biological activity of enzymes is influenced by various physiological conditions, often referred to as ‘microenvironments’. Extracellular ions (e.g., Ca^2+^, Mg^2+^, Zn^2+^) play an important role as modulators of activity of enzymes [23,24]. Maintenance of structural and functional stability in tissue or fluid compartments is therefore critical for therapeutic efficacy. The aim of this study was to investigate the influence of various conditions and biological matrices on the bactericidal activity of P128 preliminary to its consideration for parenteral use in humans.

The primary criteria for a therapeutic entity are safety and efficacy, as judged by lack of any form of toxicity when administered along with desired biological activity. Cytotoxicity of antimicrobial peptides is one of the main reasons that prevent the use of this class of molecules as antibacterial drugs. Even antimicrobial peptides that are a part of innate immunity are subject to inhibition in physiological fluids and conditions and show relatively poor activity or are rapidly inactivated [10-12]. It is imperative that molecules of heterologous origin, such as P128 be thoroughly evaluated for potential effects of biological matrices and associated microenvironments on its activity and any form of toxicity the molecule may present to humans.

### P128 dose response

*In vitro* assays have to be carefully designed considering various factors, to enable results and outcomes to be extrapolated to other purposes or translated to further studies. Here, a dose response study was performed, where P128 was tested against *S. aureus* in a checkerboard assay, correlating the dose-dependence of P128 with variation in target bacterial cell numbers.

**Figure 1:**
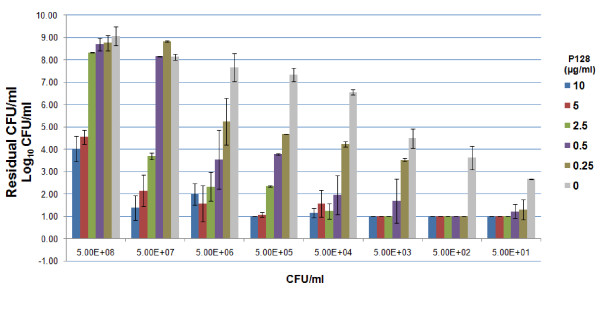
**P128 Dose Response.** Biological activity of P128 at different protein concentrations was correlated with varied target cell numbers. P128 in the range 0.25 μg/ml – 10 μg/ml was added to *S. aureus* cells taken in the range of 5×10^1^ – 5×10^8^ cells/ml. Bactericidal activity was assessed by the CFU-reduction assay. This was evaluated in duplicates and the experiment performed twice. Results are expressed as mean residual CFU/ml±standard deviation.

The results indicate that as little as 50 ng of P128 was able to kill >2 log_10_ CFU/ml (>99%, p<0.001) of 10^5^ cells. However, this dose does not cause significant reduction when 10^6^ cells were present in the same volume. In case of the higher doses tested, 500 ng of P128 was sufficient to kill >4 log_10_ CFU/ml (>99.99%, p<0.001) of 10^7^ cells and 1 μg of P128 reduced 10^8^ cells by >4 log_10_ CFU/ml (>99.99%, p<0.001). These results established the dose dependent bactericidal activity of P128 on *S. aureus* cells (Figure 1). The optimal protein concentration required to effect significant CFU reduction for a specified range of bacterial cell numbers was arrived at. The concentrations of P128 to be used for evaluation of activity under the different test conditions were judged based on these results.

### Effect of divalent cations and EDTA as modulators of P128 activity

It is well known that inorganic ions and salts are capable of influencing the biological activity of enzymes. The activity of several bacteriolytic enzymes is modulated by different ion species. Bacteriophage endolysin LysH5 shows enhanced activity in the presence of Ca^2+^ and Mg^2+^[25]. Other reported bacterial cell wall degrading enzymes have been shown to be dependent on the presence of Ca^2+^ or Zn^2+^ for activity [26,27], or inhibited by one or other ion species such as Mn^2+^[25,28]. Zn^2+^ dependent bactericidal activity against Gram-positive bacteria is exhibited by cell wall degrading enzymes, including lysostaphin which is a zinc-containing metalloprotease [29].

Staphylolytic endopeptidases have been evaluated for EDTA inhibition of activity [30] and lysostaphin has been reported to be inhibited by EDTA [31].

**Figure 2:**
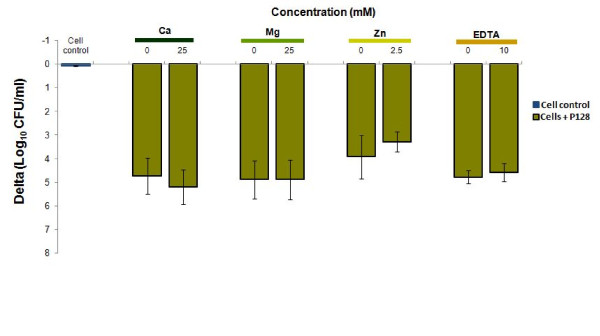
**Effect of divalent cations and EDTA as modulators of P128 activity.** 2 μg of P128 (10 μg/ml) was pre-incubated at 37°C for 30 min in concentrations of upto 50 mM of CaCl_2_ or MgCl_2_; 5 mM Zinc Acetate; 20 mM K_2_ EDTA before being tested on *S. aureus* cells. The final concentrations in the assay were 25 mM of CaCl_2_ or MgCl_2_; 2.5 mM Zinc Acetate; 10 mM K_2_ EDTA. P128 was lethal to *S. aureus* cells in the presence of millimolar concentrations of EDTA or divalent cations; Ca^2+^, Mg^2+^ and Zn^2+^. This was evaluated in duplicates and the experiment performed thrice. Results are expressed as mean residual CFU/ml±standard deviation.

We tested the effect of calcium, magnesium and zinc on the bactericidal activity of P128 to study whether concentrations of these ions would inhibit or enhance antibacterial activity of P128. All P128 treatments led to a significant reduction (>3 Delta log_10_ CFU/ml, >99.9%, p<0.001) in *S. aureus* CFU. This activity was maintained in the absence or presence of millimolar concentrations of all the three divalent cations. The highest concentration we tested was 25 mM in case of CaCl_2_ and MgCl_2_ and 2.5 mM in case of Zinc Acetate (Figure 2). Irrespective of the presence of the cation, there was no significant difference in activity within each assay condition. We conclude that while none of the ions tested potentiated the biological activity of P128, there was no loss in bactericidal activity observed. This is encouraging since the concentrations of ions tested here is higher than present in the physiological context. To confirm that soluble magnesium, calcium or zinc is not essential for enzymatic activity of P128, we tested the effect of EDTA, which is an ion-chelator and used to inhibit cation-dependent enzymes. There was a significant reduction (>4 Delta log_10_ CFU/ml, >99.99%, p<0.001) in CFU (Figure 2) in the presence of millimolar concentrations of EDTA (upto 10 mM) showing no loss of activity.

### P128 activity in human plasma, serum and whole blood

We studied the bactericidal activity of P128 in biological fluids in an attempt to simulate the milieu in which P128 could encounter the pathogen *in vivo*. Strong antimicrobial activities in artificial media or buffer systems *in vitro* may not be reproducible in complex fluid matrices such as whole blood, plasma, and serum that contain multifactorial components [32]. Additionally, whole blood and other fluid biomatrices may contain peptidases which could potentially degrade proteinaceous molecules, such as P128. The lethal outcome of a peptidoglycan degrading enzyme activity relies upon the occurrence of osmotic lysis when patches of the cell wall are broken down [33]. Complex matrices such as blood may provide osmotically stable environments which could prevent bacterial cell lysis. Such conditions could favor target pathogenic bacteria and thereby reduce their susceptibility to P128. Human sera has been previously reported to have an antagonizing effect on the antibacterial activity of lysostaphin [34]. On the other hand, it is also reported that blood and blood fractions may contain components that complement or potentiate the action of antimicrobial agents resulting in amplified pathogen killing [35].

Based in part on these concepts, we evaluated the antistaphylococcal activity of P128 in blood and blood fractions.

**Figure 3:**
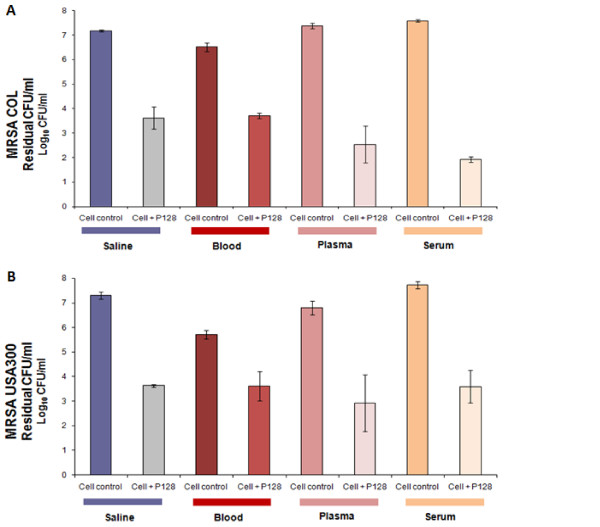
**Activity of P128 in biological matrices.** P128 demonstrates potent bactericidal effects against MRSA clinical isolates (**A**) COL and (**B**) USA300 in the presence of human biological matrices - whole blood, plasma and serum. Both experiments were performed twice. Results are expressed as mean residual CFU/ml±standard deviation.

Interestingly, no inhibitory effect was observed on P128 activity under the test conditions due to the components in any of these biological fluids. There was some antimicrobial effect observed in the case of *S. aureus* USA300 treated with whole blood. However, this did not potentiate the antibacterial activity of P128. P128 reduced staphylococcal CFU by >2 log_10_ CFU/ml (>99%, p<0.01) in serum, plasma and blood, which was comparable to the reduction seen in saline, which was used as a control matrix (Figure 3). No lysis of red blood cells (RBCs) was noted (data not shown), indicating absence of erythrolytic toxicity.

This favorable behavior of P128 under the tested conditions is of direct relevance to the potential therapeutic applications of the protein.

### Effect of hyper-immune sera on P128 activity

One of the potential challenges to the use of heterologous antibacterial proteins for therapy is elicitation of humoral immune response which could reduce or completely block its antibacterial activity, especially if to be administered repeatedly in the course of treatment. However, only a fraction of the antibodies elicited by a complex macromolecular immunogen which possesses biological activity is likely to be directly involved in the neutralizing of that activity. The inhibitory antibodies would be those directed against a limited number of antigenic epitopes localized within the catalytic site or the biologically active conformation of the protein. Sera with high antibody titers (Ab titer of 10240) to lysostaphin were able to neutralize lysostaphin activity *in vitro*[21]. The effect of hyper-immune serum is reported with other phage derived antibacterial proteins without loss of antibacterial activity [36,37]. However, even if an inhibitory effect had occurred, it would have been overcome by the high concentration of protein that was used in these studies.

In this work, we used hyper-immune sera raised against P128 in rabbits and evaluated the effect of the sera on the bactericidal activity of P128.

**Figure 4:**
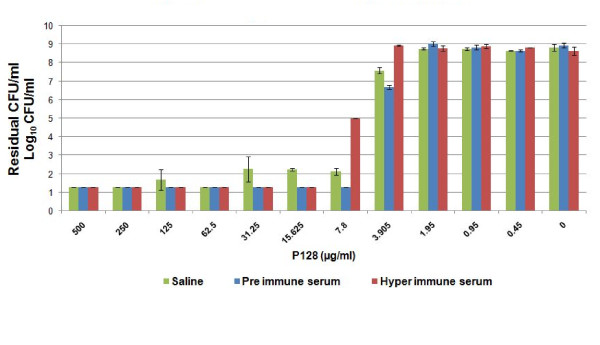
**Effect of hyper-immune sera on P128 activity.** P128 was exposed at varying concentrations to hyper-immune serum and tested for biological activity against 10^8^ cells of MRSA USA300 in the continued presence of the serum. The experiment was performed twice. Results are expressed as mean residual CFU/ml±standard deviation.

The ELISA titre of P128 antibody was >1:100,000 (data not shown). As seen in Figure 4, a minimum concentration of 3.9 μg/ml of P128 reduced cell numbers of *S. aureus* USA300 by >1 log_10_ CFU/ml (>90%, p < 0.05) in case of both pre-immune sera and saline. This correlated with the retention of activity of P128 in normal rabbit serum. In case of hyper-immune sera, the minimum concentration of P128 required for >2 log_10_ CFU/ml reduction in viable cells was 2-fold higher than for pre-immune sera, which we do not consider to be of significance in this immune-assay format. Therefore, P128 retained activity in saline, pre-immune sera or hyper-immune sera. The high affinity and rapid binding of P128 to the staphylococcal peptidoglycan could be responsible for the lack of inhibition by antibodies, which is very encouraging in the view of its proposed use against systemic infection.

### *In vitro* cytotoxicity of P128

**Figure 5:**
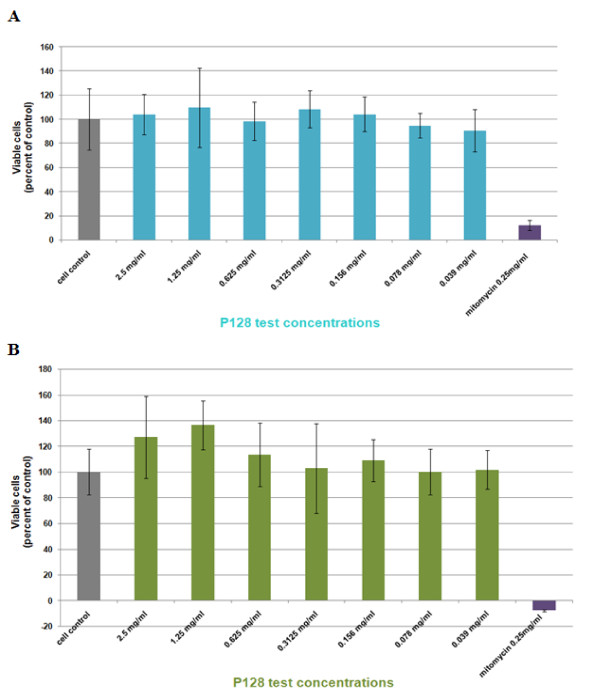
***In vitro *cytotoxicity of P128.** Cytotoxicity of P128 was evaluated on mammalian cell lines (**A**) Vero cells and (**B**) HEp2 cells. Mitomycin C was used as a control cytotoxic agent and absorbance at 590 nm was interpreted as a function of cell viability. Calculation of viable cells in case of the Mitomycin C or P128-treated groups was based on the cell-control and taking viability in the control group to be 100%. Each group was evaluated in quadruplicates and the whole experiment repeated twice. Results are expressed as means±standard deviation.

Antimicrobial peptides and proteins are known to be a part of the innate defense system in humans [38]. Often many of these, show optimal antimicrobial functions only if their distribution is restricted to specific environments and under such conditions, host toxicity is also minimized [39]. It has been shown that changes in metabolic activity are better indicators of early cell injury. Use of respiratory indicator dye MTT to measure mitochondrial activity as an indicator of metabolic function is a simple method that is accepted as a routine cytotoxicity test [40]. In the course of development of P128 as an antistaphylococcal drug for human use, P128 was tested on one normal cell line, Vero and a carcinoma cell line, HEp2 (Figure 5). Cells were treated with P128 in the range from 0.039 mg/ml to 2.5 mg/ml using the MTT colorimetric assay. Mitomycin C, used as control toxic agent at 0.25 mg/ml reduced the number of viable cells to less than 20% (p < 0.05) in both cell lines. P128 was found to be non-toxic to both HEp2 and Vero cells up to the highest concentration of 2.5 mg/ml tested. Typically, the concentration of test agent that reduces cell viability to 50%, referred to as CC_50_ or 50% cytotoxic concentration is calculated in comparison to control cells. For P128, no reduction in viability of cells could be observed at the highest concentration tested, which is 2.5 mg/ml and which corresponds to more than 100 times the MIC of this protein for most *S. aureus* isolates [20]. Therefore, absence of *in vitro* cytotoxicity as tested on immortalized somatic cells can be accepted as an early indicator of safety of P128, paving the way for further characterization and preclinical safety/toxicity studies in animals.

## Conclusions

P128 exerts potent and remarkably durable antimicrobial activity as evidenced by comparable bactericidal activity in conventional artificial matrices, such as buffers and saline and in complex matrices or biological fluids including whole blood, plasma and normal and hyper-immune sera.

The antistaphylococcal activity of P128 was not influenced by the presence of divalent cations, Ca^2+^, Mg^2+^ or Zn^2+^ & EDTA.

The protein retains its antistaphylococcal, bactericidal activity under physiological conditions that are likely to be relevant to its therapeutic use *in vivo*. P128 did not show any cytotoxic effects on mammalian cells.

## Authors’ contributions

BS and SEG participated in study design, coordination and contributed to data interpretation. RC, MD and AAJ participated in P128 dose response, modulator studies and testing in biological matrices along with SEG. UPT and SNM evaluated the *in vitro* cytotoxicity of P128. SEG helped draft the manuscript. All authors read and approved the final manuscript.

## Competing interests

The authors declare they have no competing interests.
